# COVID-19 in the Federation of Bosnia and Herzegovina: Strengthening the public health surveillance using a web-based system to inform public health response, March 2020–March 2022

**DOI:** 10.17305/bb.2022.8050

**Published:** 2023-08-01

**Authors:** Sanjin Musa, Mia Blažević, Ariana Kavazović, Jasmin Durmišević, Siniša Skočibušić

**Affiliations:** 1 Institute for Public Health of the Federation of Bosnia and Herzegovina, Sarajevo, Bosnia and Herzegovina; 2 Sarajevo School of Science and Technology, Sarajevo, Bosnia and Herzegovina; 3 Institute for Health and Food Safety Zenica, Zenica, Bosnia and Herzegovina; 4 Faculty of Medicine, University of Mostar, Mostar, Bosnia and Herzegovina

**Keywords:** Coronavirus disease (COVID-19), surveillance, response measures, Federation of Bosnia and Herzegovina (FBiH), Bosnia and Herzegovina

## Abstract

In response to the significant public health threat caused by coronavirus disease (COVID-19), real-time surveillance, containment, and mitigation measures were implemented in the Federation of Bosnia and Herzegovina (FBiH). Our objective was to describe the surveillance methodology, response measures, and epidemiology of COVID-19 cases in FBiH from March 2020 to March 2022. The surveillance system implemented across FBiH enabled health authorities and the population to monitor the development of the epidemiological situation, the daily number of reported cases, as well as basic epidemiological characteristics and geographic distribution of cases. As of 31 March 2022, 249,495 cases of COVID-19, and a total of 8845 deaths were recorded in FBiH. Upkeeping of real-time surveillance, maintaining non-pharmaceutical interventions, and speeding up the vaccination roll-out were paramount for controlling COVID-19 in FBiH.

## Introduction

In December 2019, a novel coronavirus disease (COVID-19) emerged from Wuhan City, Hubei Province, China [1]. Several of the initial cases were traced to the Huanan Seafood Wholesale Market and identified using surveillance mechanisms that were developed during the 2003 severe acute respiratory syndrome (SARS) outbreak [2]. Initial cases presented lower respiratory infection-like symptoms, in addition to headache, dizziness, feelings of weakness, vomiting, and diarrhea [3]. Given the high homology with SARS, the new coronavirus was named severe acute respiratory syndrome coronavirus 2 (SARS-CoV-2) [3]. Since December 2019, SARS-CoV-2 has spread globally with a reproduction rate of approximately 2.2–3.3 and was officially declared a pandemic by the World Health Organization (WHO) on 11 March 2020 [4, 5].

The first cases of SARS-CoV-2 in Europe were reported on 24 January 2020 in France [6]. This prompted public health leadership across Europe to prepare for the inevitable spread of the virus to their countries. The government of the Federation of Bosnia and Herzegovina (FBiH), one of two entities comprising Bosnia and Herzegovina (BiH), reacted to the WHO statement and declared the control of COVID-19 of vital importance to FBiH on 31 January 2020. This led to the immediate formation of a crisis headquarters in the Federal Ministry of Health, as well as 10 cantonal (district) crisis headquarters. Cases spread rampantly across Europe; the first case in Bosnia and Herzegovina was reported on 5 March 2020 in Banja Luka in the Republika Srpska (RS) entity [7]. The first case in the FBiH, linked to a traveler returning from Italy, occurred on 9 March 2020 in Zenica. Here, we present the surveillance methodology, response measures, and key epidemiological characteristics of COVID-19 in FBiH.

## Materials and methods

### Surveillance methodology and data collection

Using the WHO definitions for suspected, probable, and confirmed cases of COVID-19 as a guide, the Institute for Public Health of the FBiH established a case and contact definition for the FBiH and recommendations for testing in January 2020, which were subsequently updated in February, March, August, and December 2020 [8]. At the beginning of the pandemic, an epidemiologist determined whether testing should be performed based on an epidemiologic assessment; later, the assessment of whether testing was appropriate or not was made by a general practitioner or a family physician. If testing was performed, a sample from the upper respiratory tract was then sent to an accredited laboratory in the FBiH that was designated to perform an SARS-CoV-2 specific real-time RT-PCR. Eighteen laboratories in total, both public and private, became designated to handle test results in the FBiH.

**Map 1.**
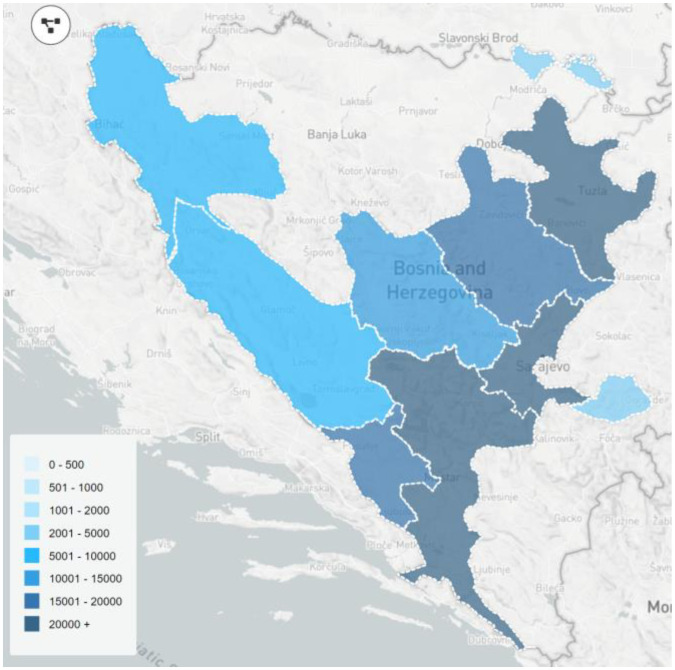
Geographical distribution of COVID-19 cases in FBiH (as shown on https://www.covid-19.ba on 31 March 2022). COVID-19: Coronavirus disease; FBiH: Federation of Bosnia and Herzegovina.

The federal web-based real-time database designed to track data on both active and cured COVID-19 cases, deaths due to COVID-19, and PCR tests conducted in the FBiH, was launched on 27 March 2020. The platform was designed through a collaboration between the Institute for Public Health of the FBiH and an outsourced information technology company. The platform was based on reporting forms according to federal legislation on infectious diseases surveillance, reporting forms based on WHO recommendations for COVID-19 reporting (8), and established influenza surveillance reporting procedures. Data collected include: demographic information (sex, age, and place of residency), date of symptom onset, date of diagnosis, clinical severity, comorbidities, hospitalization, and clinical outcome, reported by district public health institutes and accredited laboratories. Laboratory and district public health staff enter the case information in the database. The system administrator, the Department of Epidemiology of the Institute for Public Health of the FBiH, in addition to designated laboratories and district public health institutes, are the only beneficiaries with privileges to view, import, and export patient data under their administrative jurisdiction. All accredited laboratories were required to enter the data on the results of the tests performed the previous day by 12 AM through the platform. District public health institutes were also required to enter data on individuals who have recovered from COVID-19, and deaths caused by COVID-19 by 12 AM. Analysis and evaluation of these data are conducted by the Department of Epidemiology at the Institute for Public Health of the FBiH. The website, *COVID-19.ba,* visualizes data, and is available to the public. Data visualization features include a map of cases by district and municipality (Map 1), epidemic curves, and other charts and graphics, stratified by district, age, and sex. The web application runs on the Centos 7 Linux platform, utilizing PHP as the core language, in combination with HTML5, CSS3, and JavaScript for a better user experience.

### Statistical analysis

In this paper, variables were described using frequencies, percentages, and rates. The standard definition of a case fatality ratio (CFR) was used. An effective reproduction number (*R_t_*) was estimated using the 7-day moving average of new cases of COVID-19 in FBiH according to the R package “EpiEstim.” A chart of the *R_t_* with 95% intervals was created using the Shiny R web app platform [9]. Our research team made a formal review of COVID-19-related documents in FBiH in order to implement appropriate restrictive measures.

## Results

### Epidemiology

All designated institutions regularly reported data. Four private laboratories in the Sarajevo Canton did not report all detected cases to the FBiH surveillance system during April 2021; these data were subsequently reported. When the Omicron variant was at its peak, antigen-detecting rapid diagnostic tests (Ag-RDTs) were widely used. However, these data were not reported. Depending on whether there was a resurgence of cases, test and case reporting could be delayed by up to three days. In May 2021, after evaluation, two cantonal public health institutes reported 225 deaths among previously recorded COVID-19 cases (confirmed by Ag-RDTs). Data quality in terms of completeness of clinical severity, comorbidities, and hospitalization data was poor (instead of aggregated data on hospitalization was reported, and was not included in this analysis).

**Table 1:** Characteristics of COVID-19 cases and deaths in FBiH until 31 March 2022

	**Number of cases**	**Case rate per 100,000**	**%**	**Number of deaths**	**% (CFR)**
*Age groups*					
0–9	6328	289	2.5	0	0 (0)
10–19	17,864	816	7.2	3	0 (0)
20–29	30,839	1408	12.4	32	0.3 (0.1)
30–39	44,318	2023	17.8	65	0.7 (0.1)
40–49	42,343	1933	17	187	2.1 (0.4)
50–59	41,506	1895	16.6	693	7.8 (1.7)
60–69	36,646	1673	14.7	2099	23.7 (5.7)
70–79	19,638	897	7.9	2999	33.9 (15.3)
80+	9942	454	4	2762	31.2 (27.8)
Total	249,424**	11,389	100	8845	100 (3.5)
*Sex*					
Male	123,181	3,130.2	49.4	5157	58.3 (4.2)
Female	126,243	2,867.1	50.6	3688	41.7 (2.9)
Total	249,424**	5,997.2	100	8,845	100

**There are 71 cases where age and sex data are missing. CFR: Case fatality rate; COVID-19: Coronavirus disease; FBiH: Federation of Bosnia and Herzegovina.

As of 31 March 2022, there was 249,495 confirmed cases of COVID-19 in FBiH. The majority of these cases were among individuals aged 30–59 years (51.4%) (Table 1). Among the 10 districts of the FBiH, the Herzegovina–Neretva district had the highest incidence of 23,384 cases per 100,000 people, whereas the Bosnian–Podrinje district had the highest death rate of 551 per 100,000 people. There were no deaths among individuals aged 0–9 years. Cases in individuals aged 70–79 years accounted for only 7.9% of cases, but were responsible for most deaths at 33.9%. The CFR increased with age; group aged 80 and older had the highest CFR at 27.8%. FBiH had an overall CFR of 3.5%, primarily attributed to low testing capacities in certain districts, which ranged from 21,927 tests per 100,000 to 107,014 tests per 100,000 (Table 2), but also due to underdetection of asymptomatic and mild cases. The distribution of cases between males and females was relatively equal: 49.4% of cases were male and 50.6% were female (Table 1). However, the majority of deaths were male (62.3%). As of 31 March 2022, 1,248,404 COVID-19 PCR tests were performed (Table 2).

Out of 2157 (17.3%) cases documented by 31 August 2020 with reported disease severity status, 59.9% of cases experienced mild disease severity, 22.9% of cases were asymptomatic, 15.1% of cases were in severe condition, and 2.2% of cases were in critical condition (Table 3).

**Table 2:** Geographical distribution by cantons of COVID-19 cases, deaths, and tests performed in FBiH until 31 March 2022

**Canton**	**Number of cases**	**Case rate per 100,000**	**Number of deaths**	**Death rate per 100,000**	**Cumulative number of PCR tests**	**Test per 100,000**
Una-Sana Canton	9458	3530	761	284	67,151	25,068
Posavina Canton	3514	8499	153	370	9066	21,927
Tuzla Canton	42,734	9738	1857	423	206,754	47,117
Zenica-Doboj Canton	18,227	5087	1695	473	106,780	29,803
Bosnian-Podrinje Canton	2724	11,822	127	551	19,044	82,653
Central Bosnia Canton	12,538	5017	841	336	89,010	35,621
Herzegovina-Neretva Canton	50,737	23,384	874	403	218,895	100,887
West Herzegovina Canton	16,440	17,604	395	423	55,541	59,475
Canton Sarajevo	85,370	20,302	1932	459	449,988	107,014
Canton 10	7753	9690	210	262	26,175	32,717
FBiH	249,495	11,391	8845	404	1,248,404	57,002

COVID-19: Coronavirus disease; FBiH: Federation of Bosnia and Herzegovina.

**Figure 1.**
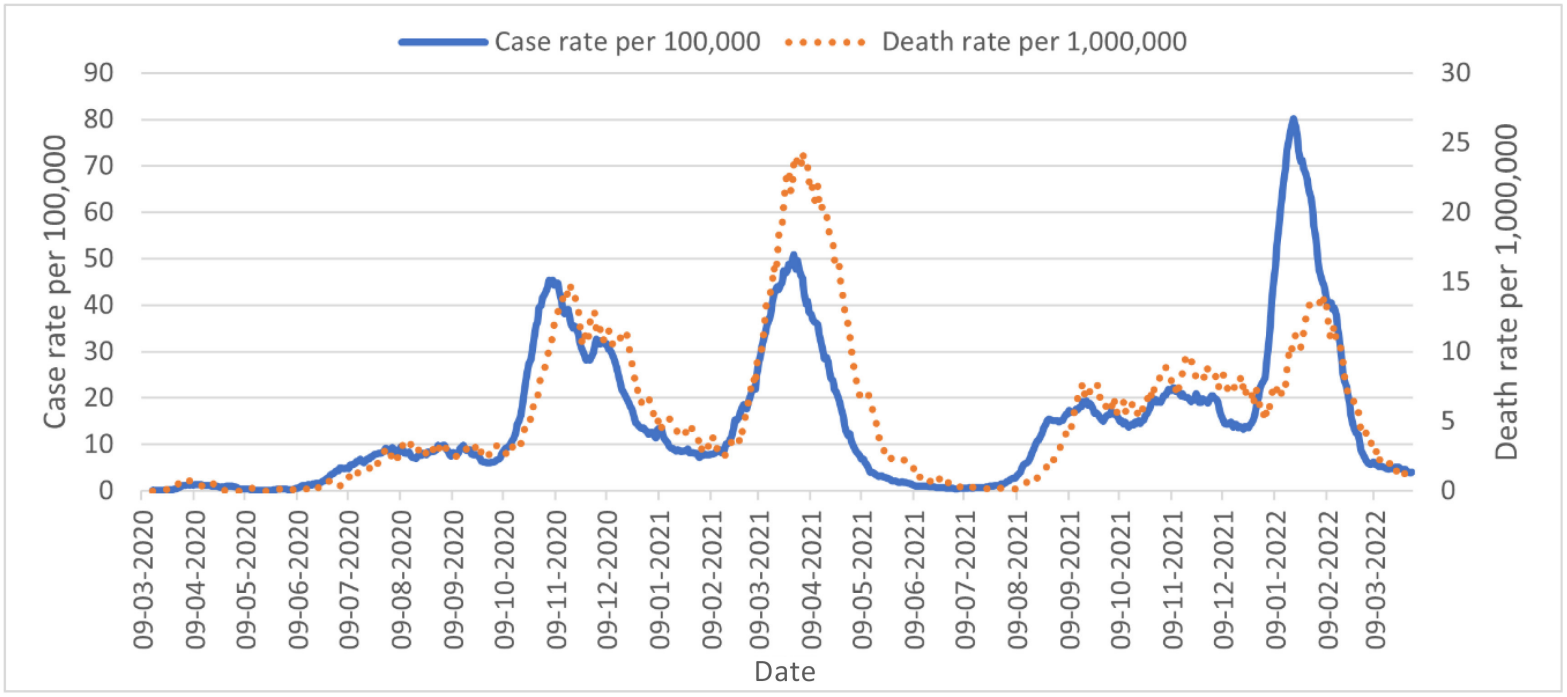
**The 7-day moving average of case rates per 100,000 and death rates per 1,000,000 in FBiH, March 2020–March 2022.** FBiH: Federation of Bosnia and Herzegovina.

Stringent preventative measures were implemented following the initial identification of COVID-19 cases in FBiH. Upon the relaxation of these measures, there was a surge in newly confirmed cases in June and July 2020 in Sarajevo and Tuzla cantons. Other districts, especially in the southern part of FBiH, experienced significant surges in August 2020. Two waves of infection were registered during autumn and winter of 2020 and spring of 2021 (Figure 1), with a 7-day moving average case rate of 45.4 cases per 100,000 people in the first week of November 2020, and 50.8 cases per 100.000 people in the last week of March 2021. Cases of COVID-19 increased again during autumn 2021 with more contagious Delta variant transmission, with a peak average case rate of 20 cases per 100,000 people. Another major surge in cases occurred between January and February 2022, with a peak average case rate of 80 cases per 100,000 people, correlating with increased Omicron variant transmission in the region. High mortality followed these incidence peaks.

**Figure 2.**
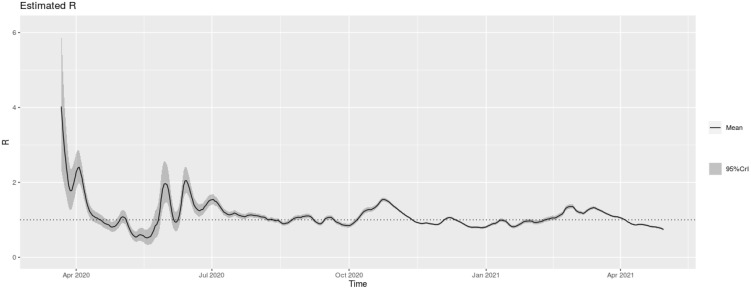
***R_t_* (95% CI) of COVID-19 in FBiH (using 7-day moving average of new cases), March 2020–April 2021.** *R_t_*: Reproduction number; COVID-19: Coronavirus disease; FBiH: Federation of Bosnia and Herzegovina. CI: Confidence interval.

During the first year of pandemic, the *R_t_* rose above 1 at the end of May, June, July, August, October 2020, February 2021, and also in the first half of March 2021, reflecting the increased number of new infections (Figure 2).

### Response measures

**Figure 3.**
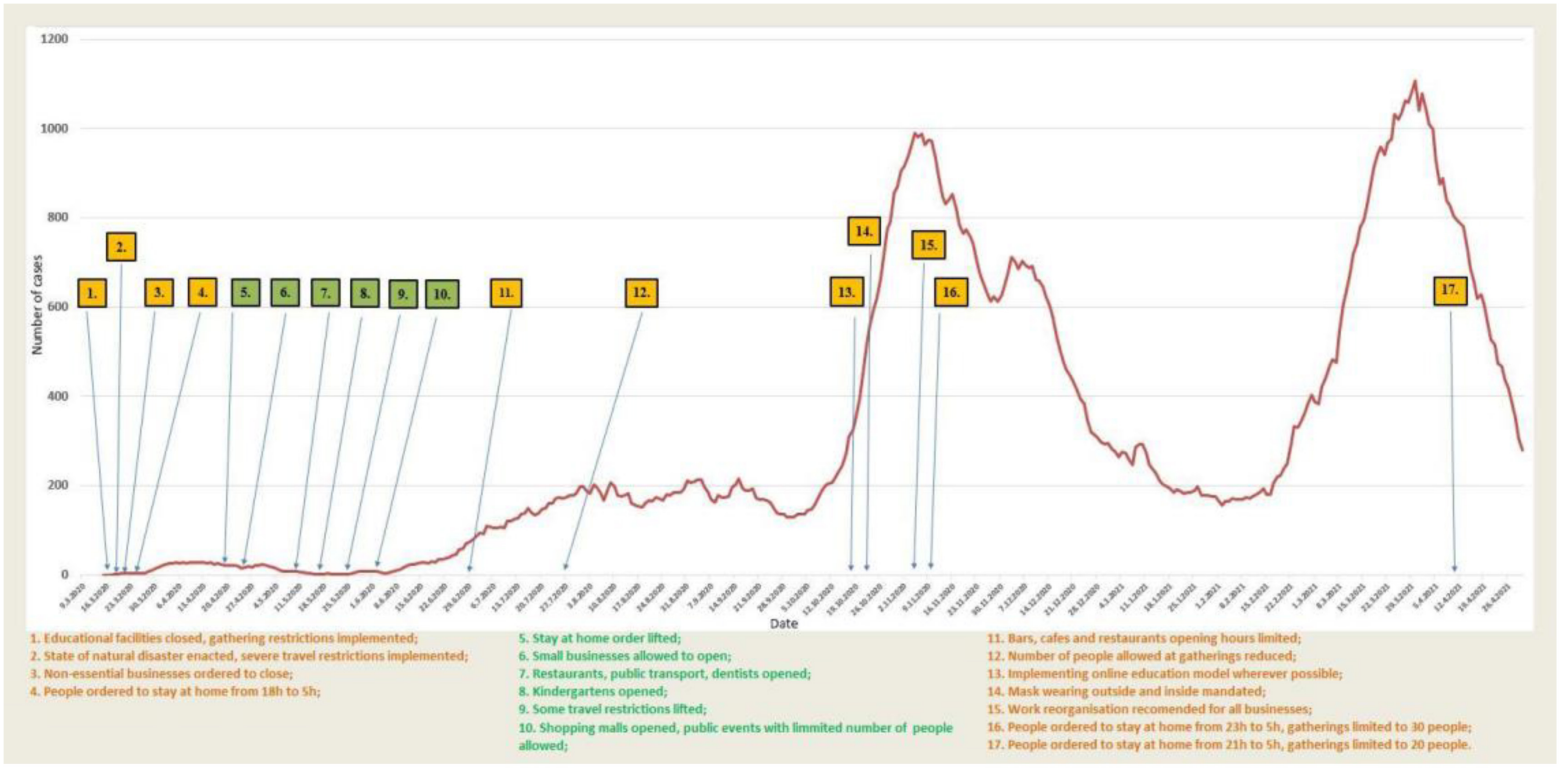
**The 7-day moving average of new cases from the day the first case was identified in FBiH, including dates of primary control measure implementation imposed at the federal level, and subsequent easing noted, March 2020–April 2021.** FBiH: Federation of Bosnia and Herzegovina.

On 12 March 2020, the crisis headquarters of the Federal Ministry of Health enacted their mitigation measures by closing all educational facilities and canceling all sports and cultural events (Figure 3) [10].

**Table 3:** Characteristics of COVID-19 cases by severity status in FBiH until 31 August 2020

**Disease severity status**	**Number of cases**	**%**
Asymptomatic	493	22.9
Mild	1291	59.9
Severe	326	15.1
Critical	47	2.2
Total	2157	100

COVID-19: Coronavirus disease; FBiH: Federation of Bosnia and Herzegovina.

A state of natural disaster was declared on 16 March 2020 by the government of FBiH, and the crisis headquarters of the Federal Department of Civilian Protection led the response until the end of May 2020 when the state of natural disaster ended. On 17 and 19 March 2020, all mass participation public events were canceled, with restaurants, bars, cafes, hair salons, and similar non-essential businesses being closed [11]. Stores selling food and household necessities, as well as soup kitchens and takeaway services were allowed to be open. On 21 March 2020, a curfew was imposed from 6 PM to 5 AM (later reduced to 8 PM to 5 AM on 29 March) and on 23 March, physical distancing was made mandatory in public spaces. A 24-h stay-at-home order was implemented for those under the age of 18 and over the age of 65, and was enforced until 14 May 2020. In April 2020, the mandatory self-isolation which was implemented in mid-March, was temporarily replaced by quarantining in designated facilities for BiH citizens entering the country; all non-citizens were banned from entering BiH. The wearing protective of masks and curfew were active until 24 April 2020.

The reopening of FBiH was implemented in phases as cases began to decrease, starting on 28 April 2020 with small businesses. Restaurants, public transport, and private dental offices began opening on 14 May 2020. On 18 May 2020, kindergartens began opening. Mandatory self-isolation for citizens of BiH entering the country was lifted on 20 May 2020, after it was initiated by authorities in RS. The final phase started on 28 May 2020, with the opening of shopping malls, and permitting public gatherings of 100 people indoors and 300 people outdoors. District crisis headquarters were allowed to implement additional restrictive measures. Along with the easing of measures, the Department of Epidemiology of the Institute of Public Health of FBiH issued a series of supplementary infection control recommendations (for example, for public transport, hotels, museums, galleries, cinemas, etc.). Upon the reopening, FBiH has seen a gradual increase in cases, rising above 100 cases per day in a 7-day average period at the beginning of July. New control measures were implemented on 26 June 2020, including limited working hours for bars, cafes, and restaurants (initially until 12 PM, later reduced to 11 PM). Public gatherings were reduced to 50 people indoors, and 100 people outdoors on 23 July 2020 [10]. Cases fluctuated between 154 and 212 cases per day during August 2020.

In autumn, when the new school year started, an online model was implemented in schools where possible, but most schools operated on a hybrid model (splitting classes, rotating classes online, and in person on a weekly or daily basis). University education was conducted online whenever possible. On 25 October 2020, a masking mandate was ordered for indoor and outdoor public spaces. During November 2020, an increase in cases warranted the implementation of stricter measures. On 10 November 2020, public gatherings were limited to 30 people inside and outside and a curfew was introduced between 11 PM and 5 AM [10]. Businesses with more than 20 employees were required to reorganize work, such as working in shifts, stricter implementation of infection control measures, and working from home wherever possible. After a gradual decrease of cases registered during December 2020 and January 2021, a new increase in cases was registered during February and March 2021. In March 2021, district crisis committees implemented additional measures according to the epidemiological situation. In the Sarajevo Canton, bars and restaurants were closed for two weeks. The Federal crisis headquarters limited gatherings to 20 people inside and outside and implemented a curfew from 9 PM to 5 AM. These new measures were maintained throughout April and were relaxed in May 2021. From May 2021 to February 2022, no major restrictive measures were implemented; in general, less stringent gathering measures were in place, and physical distancing and masking were ordered. Most measures were lifted in March 2022.

## Discussion

There have been 249,495 cases of COVID-19 and a total of 8845 deaths reported in FBiH as of 31 March 2022 (Table 2). As a response to newly emergent diseases, such as COVID-19, the availability of web-based reporting and an upkept surveillance system that integrates epidemiologic and virologic surveillance data was of critical importance for strengthening surveillance capacity and providing information for response measures, including contact tracing. In FBiH, notifiable infectious diseases must be reported according to clinical, epidemiological, and laboratory criteria guidelines from health institution or laboratories to the 10 districts public health institutes and the Institute for Public Health of the FBiH. The reporting system is paper-based (however, there is a steady transition to electronic surveillance systems, as in the Sarajevo Canton, for example) uses a standard reporting form, and is used by health professionals and laboratories.

Despite the limited capacity of the public health system in FBiH to respond to an unprecedented crisis such as the COVID-19 pandemic, the overall implementation of the surveillance system was satisfactory in terms of acceptance and timelines. All reporting health institutions reported data in the database. The electronic implementation of the surveillance system bolsters the ease of access of the system and increases the accessibility of the data to the general public. In regards to data quality, the surveillance system performs well considering the data on COVID-19 cases and deaths stratified by districts and municipalities, age group, and sex. Hospitalization, clinical severity of disease, and comorbidities are not reflected in the system.

During the period of March 2020 and March 2022, 1,248,404 PCR tests (Table 2) were performed, with significant differences in testing rates between cantons. The differences between cantonal testing rates reflect differences in testing capacities, and an underestimation of the number of COVID-19 cases. Ag-RDT tests were used during the Omicron variant surge, however, these data were not reported. A large proportion of undetected asymptomatic COVID-19 cases further contributed to an underestimation of cases.

As a middle-income country, maintaining non-pharmaceutical interventions (NPIs), such as masking mandates and social distancing measures, were key to ensuring that health systems were not overburdened [12–14]. In FBiH, after the initial implementation of NPIs during the winter and spring months of 2020, the Rt was below 1, which kept the level of transmission of COVID-19 low and allowed time to increase the health system response capacity [15, 16]; a new increase in cases was seen after the relaxation of NPIs and the re-opening of country borders. Early NPIs were implemented in both BiH entities [13]. The Rt rose above 1 during October 2020 and from February to March 2021, reflecting an increase of cases. COVID-19 case rate peaked in the first week of November 2020 and in the last week of March 2021. The implementation of additional NPIs during October and November 2020, and compliance with these measures may have been associated with a subsequent temporary reduction in COVID-19 transmission and a lower Rt.

While the benefits of maintaining restrictive measures are clear, their maintenance also causes negative economic consequences. Restrictive measures have significantly limited the way that goods are imported and exported, reduced tourism, limited productivity, and increased workplace absenteeism in Europe and beyond [17]. Loss of employment has led to the loss of income for many, leading to changes in spending behavior, and a decline in demand for a number of goods and services [17]. This can cause authorities to become hesitant to implement stricter measures out of fear of backlash from business.

The increase in incidence during February and March 2021 was presumably caused by the increased presence and increased transmissibility of the Alpha variant (previously referred to as the B.1.1.7 variant). The first cases of the Alpha variant were detected in FBiH in December; whole genome sequencing was sporadically performed [18]. Strengthening and expanding activities related to genomic sequencing should be part of a response plan. The transmissibility of the Alpha variant is estimated between 43% and 90% more transmissible than previous variants [19]. In an analysis of seven European countries, including Cyprus, Estonia, Finland, Ireland, Italy, Luxembourg, and Portugal, the Alpha variant was the most prevalent variant between week 38 in 2020 and week 10 in 2021 in all countries except Portugal [20]. The first known case of the Delta variant in the FBiH was reported on 3 July 2021 [21] The Delta variant quickly became the dominant strain in most of Europe by mid-July 2021 [22]. Although the Delta variant was reported to be approximately up to 60% more transmissible and severe than the Alpha variant, the distribution of vaccines had a substantial impact on mitigating COVID-19 epidemic in the FBiH [23]. The first known cases of the Omicron variant in the FBiH were reported in December 2021 [24]. While the symptomology associated with the Omicron variant is milder than in previous variants, the large amount of mutations on the spike protein between the Delta and Omicron variants make it much more transmissible, explaining the sharp rise in cases in FBiH between January and February 2022 [25].

The effectiveness of response measures depends on the public’s risk perception of COVID-19, and subsequent compliance with preventative measures, including vaccination. Globally, risk perception of COVID-19 is generally high, however, factors, such as trust in government, the degree of collective efficacy, and the level of adoption of protective health behaviors can lessen public risk perception [26]. As the pandemic in FBiH experienced several periods of resurgence and decline in cases, people in the FBiH may have become fatigued with maintaining preventative measures [27] The Institute for Public Health of the FBiH, with support from the WHO Regional Office for Europe, conducted a behavioral insights survey to monitor knowledge, risk perceptions, preventive behaviors, and trust to inform the pandemic outbreak response; the study suggests that high risk perception, high trust in institutions (in regards to information dissemination), and negative affect about the pandemic were associated with positive vaccination intentions [28, 29].

Given that this paper is a descriptive overview of the FBiH COVID-19 surveillance system and response, one limitation is that no modeling of any type, or time series analyses were conducted. Further modeling studies may be beneficial in assessing the impact of NPIs implementation on incidence. We lack data on how travel to and from other countries may have impacted the cumulative increase in cases during summer 2020, and what proportion of cases in FBiH is comprised of imported cases. We did not conduct an in-depth evaluation of the performance of the COVID-19 surveillance system. A representative depiction of asymptomatic cases in FBiH would require additional serological investigation [30, 31], thus a significant number of cases may be omitted from this analysis. Our research will enrich the available knowledge on the pandemic response in the country and subsequently encourage further analysis and evaluation.

## Conclusion

Real-time web-based surveillance is a vital tool that enables public health professionals and decision makers to monitor the incidence and geographic distribution of cases of newly emerging infections, such as COVID-19. The combination of well-performing real-time web-based surveillance, the use of NPIs, and faster vaccination roll-out was paramount for reducing morbidity and mortality from COVID-19; efficient response coordination, and timely communication with the public play a pivotal role in the impact of these measures. Improvements to the surveillance system made during COVID-19 pandemic can serve as a basis for further strengthening infectious disease tracking in FBiH.
